# Complement Activation Is Associated With Crescents in IgA Nephropathy

**DOI:** 10.3389/fimmu.2021.676919

**Published:** 2021-09-14

**Authors:** Zi Wang, Xinfang Xie, Jingyi Li, Xue Zhang, Jiawei He, Manliu Wang, Jicheng Lv, Hong Zhang

**Affiliations:** ^1^Renal Division, Peking University First Hospital, Peking University Institute of Nephrology, Beijing, China; ^2^Key Laboratory of Renal Disease, Ministry of Health of China, Key Laboratory of Chronic Kidney Disease Prevention and Treatment (Peking University), Ministry of Education, Beijing, China; ^3^Research Units of Diagnosis and Treatment of Immune-Mediated Kidney Diseases, Chinese Academy of Medical Sciences, Beijing, China

**Keywords:** immunoglobulin A nephropathy (IgAN), crescent, complement, lectin pathway, urinary C4d

## Abstract

**Introduction:**

Crescents, especially those found at a percentage greater than 50%, are often associated with rapid progression of kidney disease in IgA nephropathy (IgAN). The mechanism of crescents forming in IgAN is still unclear. In this study, we aimed to evaluate whether excess complement activation participates in the formation of crescents in IgAN.

**Methods:**

One hundred IgAN patients with various proportions of crescents—24 with 1%–24%, 27 with 25%–49%, 21 with 50%–74% 12 with more than 75%, and 16 without crescents—were included. Urinary concentrations of mannose-binding lectin (MBL), Bb, C4d, C3a, C5a, and soluble C5b-9 (sC5b-9) were measured at the time of biopsy. Receiver operating characteristic (ROC) curves were performed to evaluate predictive ability of renal survival for urine complement activation. In addition, historical C4d, C5b-9, and C3d were stained by immunohistochemistry.

**Results:**

IgAN patients with more than 50% crescent formation showed higher complement activation levels than the other patients (urinary C3a/creatinine (C3a/Cr): 6.7295 ng/mg, interquartile range (IQR) 1.4652–62.1086 ng/mg vs. 0.1055 ng/mg, IQR 0–1.4089 ng/mg; urinary C5a/Cr: 15.6202 ng/mg, 4.3127–66.7347 ng/mg vs. 0.3280 ng/mg, IQR 0.0859–2.4439 ng/mg; urinary sC5b-9/Cr: 98.6357 ng/mg, 8.8058–1,087.4578 ng/mg vs. 1.4262 ng/mg, 0.0916–11.0858 ng/mg, all *p*-values <0.001). The levels of urinary MBL and C4d representing lectin complement pathway showed a linear association with the proportion of crescents (r = 0.457 and 0.562, respectively, both *p*-values <0.001). Combined urine complement products could increase the predictive ability compared with crescents alone from 0.904 to 0.944 (*p* = 0.062) with borderline significance. Moreover, the glomerular C4d deposition rate elevated with the increase of proportions of crescents.

**Conclusion:**

Excess complement activation may be involved in the formation of crescents, especially diffuse crescent formation, in patients with IgAN. Urinary C4d correlated with the proportion of crescents and was a potential biomarker for disease monitoring in crescentic IgAN.

## Introduction

IgA nephropathy (IgAN) is the most common primary glomerulonephritis worldwide and is characterized by mesangial IgA deposition (1, 2). The clinical course of IgAN ranges from isolated hematuria to rapidly progressive renal failure, and the kidney biopsy findings vary from mild mesangial proliferation to diffuse crescent formation (3). Crescent formation on kidney biopsy is regarded as a prognostic indicator of poor outcomes. Those with high levels of crescents usually show rapidly progressive kidney failure and need aggressive immunosuppressive therapy (4, 5). However, to date, the pathogenesis of crescent formation in IgAN is still unclear.

Complement activation is involved in the development or progression of IgAN. Glomerular deposits of C3, properdin, C4d, mannose-binding lectin (MBL), and C5b-9 but limited C1q indicate that the alternative and lectin pathways are primarily involved in this disease (6). Complement activation can occur directly on IgA1-containing immune complexes in circulation and/or after their deposition in the mesangium, thus playing an important role in the development of IgAN (7). Glomerular deposition of C4d and plasma levels of iC3b-d were associated with the severity of the histologic lesions or clinical features (8, 9). However, to date, the roles of complement activation in the development of diffuse crescent formation in IgAN are unclear. In this study, we aimed to evaluate the extent of complement activation and its association with crescent formation.

## Patients and Methods

### Study Participants

One hundred adult IgAN patients with different proportions of crescents who were diagnosed by renal biopsy from 2004 to 2019 at Peking University First Hospital were enrolled this study: 24 patients with a proportion of crescents of 1%–24%, 27 patients with 25%–49%, 21 patients with 50%–74%, 12 patients with more than 75%, and 16 patients without crescent formation. Plasma samples were obtained from 29 of 100 patients. An additional 41 patients from whom plasma samples were collected in the same period were enrolled for the plasma complement analysis (**Supplementary Table 1**). IgAN was diagnosed by immunofluorescence showing the presence of at least 1+ (range, 0–3) IgA mesangial deposits as the dominant or codominant immunoglobulin in the mesangial deposits and the deposition of electron-dense materials in the mesangium on ultrastructural examination. Renal biopsies were scored by two pathologists blinded to the clinical data, with any differences in grading resolved by viewing the slide together under a two-headed microscope. Patients with serological or clinical evidence of other renal damage, such as systemic lupus erythematosus, vasculitis, anti-glomerular basement membrane (anti-GBM) disease, and Henoch–Schönlein purpura, were excluded. Patients with other autoimmune diseases or pregnancies were also excluded. A crescent was defined as extracapillary proliferation of more than two cell layers of any size; a cellular crescent was defined by >50% of the lesion occupied by cells; a fibrocellular crescent was defined by ≤50% of the lesion occupied by cells and <90% occupied by matrix; a fibrous crescent was defined by ≥90% matrix composition. Clinical characteristics, including age, sex, serum creatinine, 24-h protein excretion, blood pressure, and complement C3 and C4 levels, were collected at the time of renal biopsy.

The study was approved by the ethics committee at the Peking University First Hospital. Informed written consent was obtained from all participants.

### Detection of Activated Complement Components

Urine and plasma samples were collected from the patients before renal biopsy, and samples from the healthy controls were stored at −80°C until analysis. Activated complement components, including Bb, C4d, C3a, C5a, and SC5b-9, were detected by ELISA kits (Quidel, USA). Urinary levels of MBL were detected by ELISAs using a commercial MBL Oligomer ELISA kit (Bioporto, Hellerup, Denmark). All assays were conducted according to the manufacturer’s instructions. Urine and plasma samples had only one freeze/thaw cycle before analysis. Frozen specimens were thawed rapidly at 37°C and immediately moved to ice to prevent complement activation prior to dilution. After dilution, the samples were loaded into the microassay wells as rapidly as possible.

### Immunohistochemistry for C4d, C3d, and C5b-9

To study the deposition of complement activation products in kidneys of IgAN patients with different proportions of crescents, complement activation products C4d, C3d, and C5b-9 were detected by immunohistochemistry. Ten patients with 0%–24% crescents, 10 patients with 25%–49% crescents, and 10 patients with over 50% crescents were included. C4d, C5b-9, and C3d immunohistochemical staining was performed on 4-µm deparaffinized and rehydrated sections of formaldehyde-fixed renal tissue, using polyclonal rabbit anti-human C4d (Biomedica, Vienna, Austria) (1:50), polyclonal rabbit anti-human C3d (DAKO, Denmark) (1:1,000), and monoclonal mouse anti-human C5b-9 (DAKO, Denmark) (1:50) as the antibodies, respectively. The sections for C3d and C4d staining were treated with 0.4% pepsin (Zhongshan Golden Bridge Biotechnology, Beijing, China) 30 min for antigen retrieval. The sections for staining C5b-9 were treated with 0.5 mg/ml of proteinase K for antigen retrieval. Sections of IgAN patients previously proven C4d, C5b-9, and C3d positive and a section with antineutrophil cytoplasmic antibody (ANCA)-associated vasculitis served as the positive and negative controls, respectively. The sections were examined by light microscopy. Patients were classified as positive when >25% of the non-sclerotic glomeruli were positive for complement products.

### Prognosis End Point

For each patient, the date of renal biopsy was recorded as the baseline point. Follow-up time was considered as the interval between renal biopsy and the last outpatient visit. The primary end point of the study was the cumulative percentage of patients who developed end-stage kidney disease (ESKD) (defined by 12 or more continuous weeks of renal replacement therapy or by kidney transplantation). No patient died before the time of ESKD.

### Statistical Analysis

Non-normally distributed and normally distributed quantitative data are expressed as medians and interquartile ranges (IQRs) and means ± standard deviations, respectively. Categorical data are shown as frequencies or ratios. Student’s t-test for normally distributed data, or non-parametric test (Mann–Whitney U test) for non-normally distributed data, was used for the differences of quantitative parameters between groups. Spearman’s correlation was performed between urinary complement levels and other variables. The Kaplan–Meier curves were performed to evaluate the effect of different proportions of crescent formation on renal survival. Package “pROC” was used to create receiver operating characteristic (ROC) curves to determine the different models in prediction of renal survival. A two-tailed *p*-value <0.05 was considered statistically significant. All statistical analyses were performed using SPSS version 22.0, and graphs were generated using R version 4.0.3.

## Results

### Baseline Demographic, Clinical, and Pathological Characteristics

Overall, 100 IgAN patients with different proportions of crescents were enrolled in this study: 16 patients without crescents (C0), 24 with 0%–24% crescents (C1), 27 with 25%–49% (C2), 21 with 50%–74%, and 12 with more than 75%. There were 68 (68%) males and 32 (32%) females with a mean age at the time of kidney biopsy of 36.2 ± 12.5 years. As shown in **Table 1**, a higher proportion of crescents was associated with higher levels of plasma creatinine or proteinuria, especially in those patients with crescent more than 50% (**Table 1**).

**Table 1:** Baseline characteristics of IgAN patients with various crescents forming.

Characteristics	0% (N = 16)	0%–24% (N = 24)	25%–49% (N = 27)	50%–74% (N = 21)	75%–100% (N = 12)	Total (N = 100)	*P*-value
Male (%)	9 (56.3%)	17 (70.8%)	17 (63.0%)	17 (81.0%)	8 (66.7%)	68 (68.0%)	0.550
Age	31.8 ± 7.8	40.1 ± 11.6	39.1 ± 14.0	32.5 ± 12.0	34.2 ± 15.4	36.2 ± 12.5	0.096
SBP (mmHg)	127.4 ± 15.3	133.6 ± 21.9	133.4 ± 24.2	147.0 ± 18.0	136.3 ± 15.2	135.7 ± 20.8	0.051
DBP (mmHg)	80.9 ± 11.5	86.0 ± 13.7	82.0 ± 16.5	90.8 ± 16.6	80.6 ± 10.8	84.5 ± 14.8	0.159
Scr (µmol/L)	89.0 (71.3–136.1)	122.7 (88.1–166.6)	182.1 (94.9–333.5)	398 (289.9–511.6)	292.5 (201.8–686.8)	163.5 (97.3–375.9)	<0.001
Proteinuria (g/day)	0.9 (0.5–3.7)	1.8 (1.1–3.3)	2.4 (1.4–5.3)	5.5 (4.2–7.4)	5.4 (3.5–8.4)	3.4 (1.4–5.6)	<0.001
Albumin (g/L)	39.9 ± 3.8	36.6 ± 5.1	34.2 ± 5.5	31.7 ± 5.1	28.1 ± 7.2	34.4 ± 6.3	<0.001
IgG (g/L)	9.9 ± 3.1	10.4 ± 2.5	9.6 ± 2.7	7.5 ± 2.5	9.2 ± 5.5	9.4 ± 3.2	0.036
IgA (g/L)	3.1 ± 1.4	2.8 ± 0.8	3.4 ± 1.1	2.9 ± 1.2	2.7 ± 0.9	3.1 ± 1.1	0.267
C3 (g/L)	1.01 ± 0.31	1.01 ± 0.21	0.96 ± 0.20	0.95 ± 0.19	1.00 ± 0.24	0.98 ± 0.22	0.837
C4 (g/L)	0.21 ± 0.06	0.27 ± 0.08	0.26 ± 0.77	0.27 ± 0.53	0.27 ± 0.52	0.26 ± 0.69	0.128
Total crescents (%)	0% (0–0)	15.1 (9.5–20.5)	39.1 (30.0–52.6)	64.3 (54.8–74.0)	82.3 (78.7–86.6)	35.1 (11.8–64.3)	<0.001
Cellular+fibrocellular crescents (%)	0 (0–0)	12.1 (8.8–15.9)	33.3 (30.0–40.0)	60.0 (52.4–66.7)	81.5 (78.0–86.5)	32.5 (10.5–57.4)	<0.001
Fibrous crescents (%)	0 (0–0)	0 (0–0)	0 (0–9.10)	0 (0–6.8)	0 (0–0)	0 (0–0)	0.283

SBP, systolic blood pressure; DBP, diastolic blood pressure; Scr, serum creatinine; IgAN, IgA nephropathy.

### Association of the Levels of Urinary Complement Activation Products and Crescents

**Figure 1:**
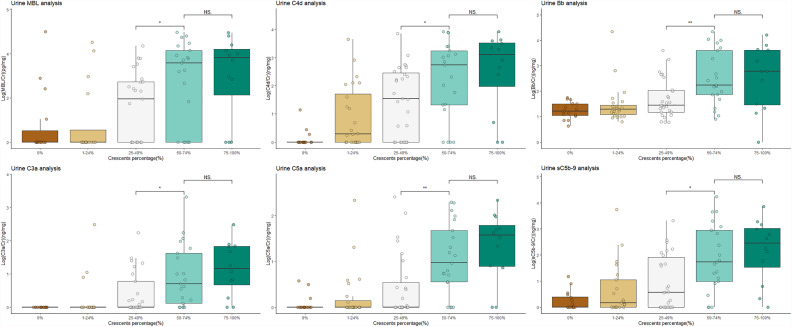
Patients with crescentic IgAN had higher levels of activated complement products in urine than non-crescentic IgAN patients. NS, not significant; IgAN, IgA nephropathy. The urine values were corrected by urinary creatinine. All the data were log10-transformed.

As shown in **Figure 1**, urinary C3a and C5a, representing the common pathway of complement activation, remained at low levels in the patients with a proportion less than 50%, while they increased significantly when the proportion of crescents reached 50% (p < 0.001). A similar trend was observed in the levels of Bb, representing the alternative pathway. The patients with a percentage of crescents >50% showed higher complement activation levels than the other patients (urinary C3a/Cr: 6.7295 ng/mg, IQR 1.4652–62.1086 ng/mg vs. 0.1055 ng/mg, IQR 0–1.4089 ng/mg; urinary C5a/Cr: 15.6202 ng/mg, 4.3127–66.7347 ng/mg vs. 0.3280 ng/mg, IQR 0.0859–2.4439 ng/mg and urinary Bb/Cr: median 0.0224 μg/mg, IQR 0.0125–0.0484 μg/mg vs. 0.2684 μg/mg, 0.0476–4.007 μg/mg, all *p*-values <0.001).

**Figure 2:**
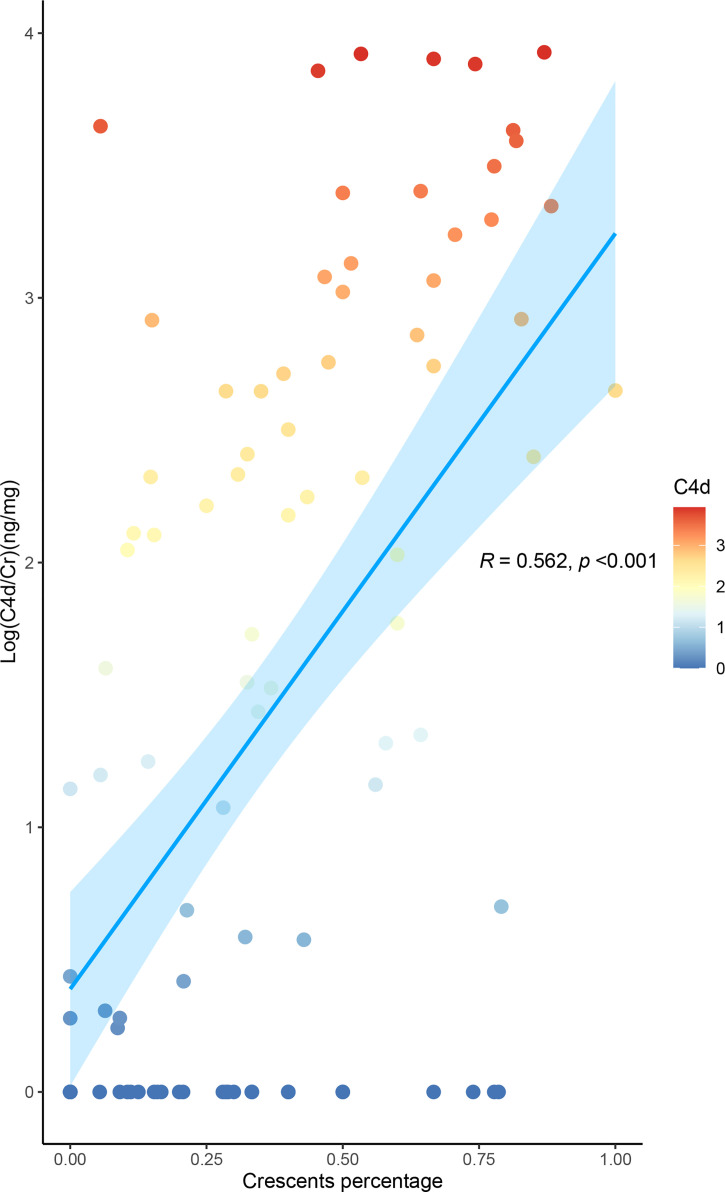
Level of urinary C4d is associated with the proportion of crescents in IgA nephropathy. The urine values were corrected by urinary creatinine. All the data were log10-transformed.

The levels of urinary C4d and MBL, representing the lectin pathway, and those of C5b-9, the terminal complement activation products, were positively correlated with the proportion of crescents in IgAN (all *p*-values for the trend <0.001). This association was most obvious in urinary C4d. The median urinary C4d/Cr value among the patients with proportions of 0%, 1%–24%, 25%–49%, 50%–74%, and >75% were 0 ng/mg (range 0–0.637), 1.9651 ng/mg (0–93.6551), 35.3148 ng/mg (0–317.9359), 552.6448 ng/mg (17.6306–2,112.9755), and 1,403.1766 (66.5081–3,724.6789, respectively (*p* for trend <0.001). The levels of urinary C4d showed a linear association with the proportion of crescents (r = 0.562, *p* < 0.001) (**Figure 2**).

### Association of Urinary Complement Activation Products and Clinical Characteristics

As shown in **Table 2**, the levels of urinary complement activation products, including urine C3a, C5a, Bb, MBL, C4d, and C5b-9, were positively correlated with serum creatinine (the values of r were 0.677, 0.655, 0.557, 0.408, 0.583, and 0.599, respectively) and proteinuria (r values were 0.652, 0.719, 0.552, 0.487, 0.547, and 0.599, respectively) (all *p*-values <0.001).

**Table 2:** Association of urinary activated complement products and baseline characteristics in patients with IgA nephropathy.

Urine complement products	Proteinuria (g/day)		Serum creatinine (μmol/L)	
	r	p	r	p
MBL/Cr	0.487	<0.001	0.408	<0.001
C4d/Cr	0.547	<0.001	0.583	<0.001
Bb/Cr	0.552	<0.001	0.557	<0.001
sC5b-9/Cr	0.599	<0.001	0.599	<0.001
C3a/Cr	0.652	<0.001	0.677	<0.001
C5a/Cr	0.719	<0.001	0.655	<0.001

Cr, creatinine; IgAN, IgA nephropathy; MBL, mannose-binding lectin.

### Immunohistochemistry for C4d, C3d, and C5b-9

**Figure 3:**
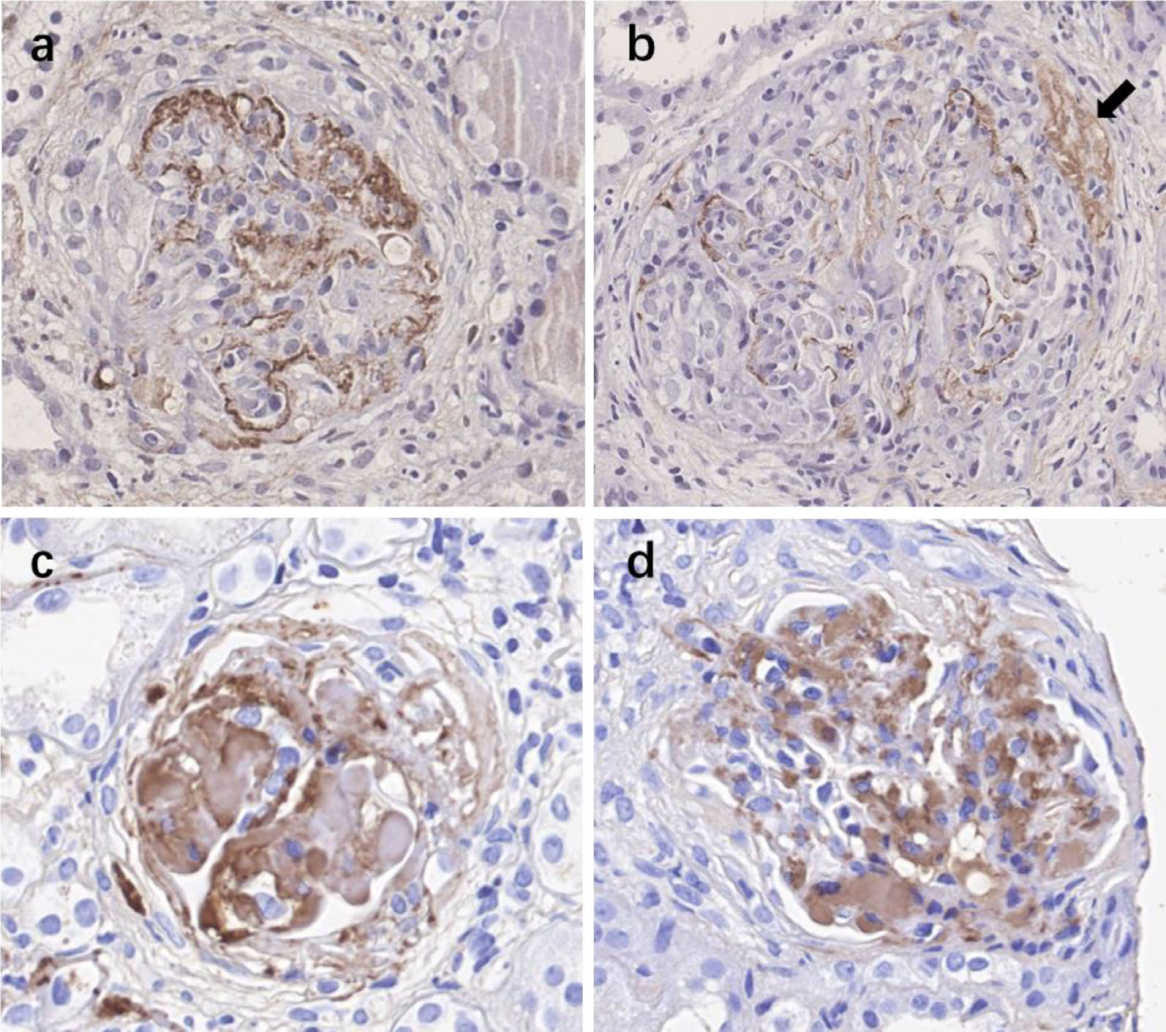
Immunohistochemistry staining for complement activation products C4d, C3d, and C5b-9 in kidney specimens of IgAN patients with various crescent formations. **(A)** Positive staining of C4d in the mesangial area of the glomerulus. **(B)** Positive staining of C4d both in the mesangial area of the glomerulus and within the crescent (black arrow). **(C)** Positive staining of C5b-9. **(D)** Positive staining of C3d. IgAN, IgA nephropathy.

A total of 30 participants including patients with crescents of 0%–24% (n = 10), 25%–49% (n = 10), and over 50% crescent formation (n = 10) were included for immunohistochemistry. Glomerular staining for C4d (>25% of glomeruli) were observed in two (20%) cases in group with crescents of 0%–24%, seven (70%) cases in 25%–49%, and all 10 cases in the groups with crescents over 50%. Positive glomerular staining was seen predominantly in the mesangial area, very often within the crescents and the sclerotic lesions. Glomerular C5b-9 deposition was observed in seven (70%) cases in the group with crescents 0%–24%, while all positive in groups with crescents >25% or >50%. Positive C3d staining was observed in all 30 patients (**Figure 3**).

### Prognosis of Patients With Different Proportions of Crescents

**Figure 4:**
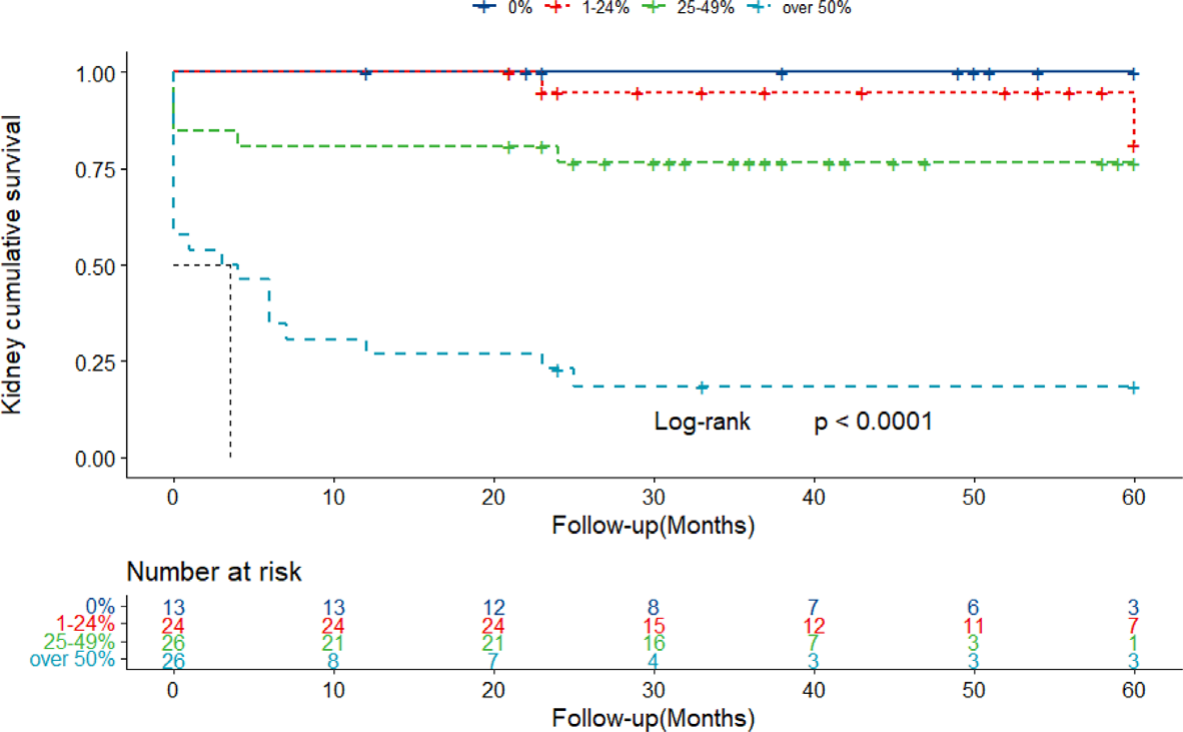
Kaplan–Meier curves of renal survival in IgAN patients with different proportions of crescents. Patients were retrospectively followed up with a mean of 29.5 months. Twenty-nine ESKD cases were collected throughout the follow-up. IgAN, IgA nephropathy; ESKD, end-stage kidney disease.

To evaluate the effect of crescent formation on kidney progression in IgAN, the Kaplan–Meier method was used to assess cumulative incidence of ESKD among patients with different percentage of crescents. According to the percentage of glomeruli involved with crescents, the patients were divided into four groups: group 1, absence of crescents; group 2, crescents less than 25%; group 3, 25%–49%; and group 4, more than 50%. During a mean follow-up of 29.5 months, 29 patients progressed to ESKD. No patient died before the time of ESKD. ESKD occurred in zero of 13 patients in group 1, in two of 24 (8.3%) in group 2, in six of 26 (23.1%) in group 3, and in 21 of 26 (80.8%) (log-rank, *p* < 0.001) (**Figure 4**).

### Assessment of the Prognostic Values of Urine Complement Activation

**Figure 5:**
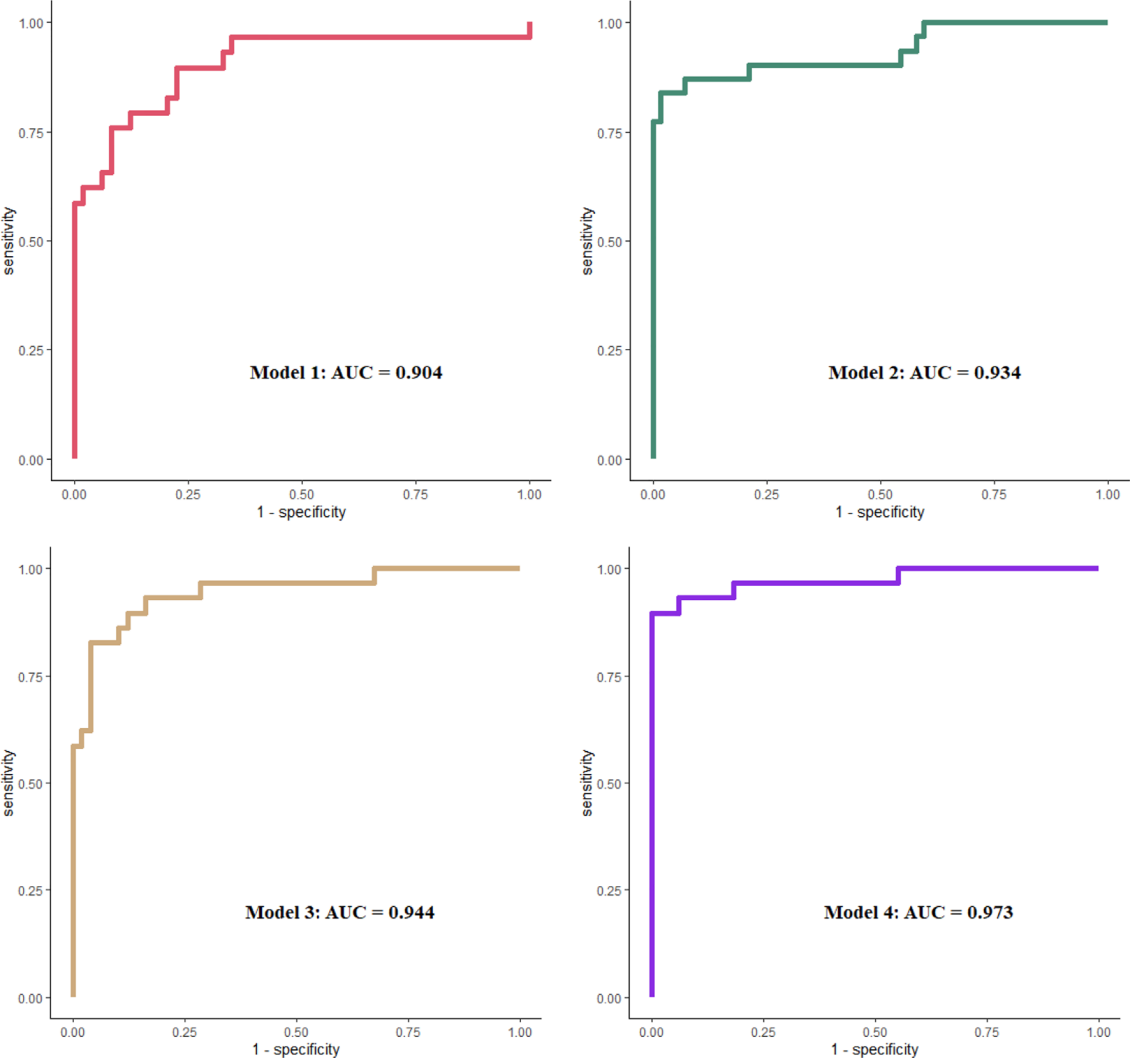
ROC curves in evaluation different models to predict ESKD. Model 1 included urinary MBL, Bb, C4d, C3a, C5a, and sC5b-9, all corrected by urine creatinine. Model 2 included Scr, UTP, and crescent percentage. Model 3 included covariates in model 1 plus crescent percentage. Model 4 included covariates in model 1 plus model 2. All covariates were analyzed as continuous data. ROC, receiver operating characteristic; ESKD, end-stage kidney disease; MBL, mannose-binding lectin; Scr, serum creatinine.

To explore the prognostic values of urine complement products, different clinical models were evaluated for their ability to predict renal survival by performing ROC curve analysis. Combined urine-activated complement products showed good performance in predicting prognosis. The area under the curve (AUC) for combined urine complement activation products (Model 1) was 0.904 (95% confidence interval [CI], 0.825–0.984). Combined urine complement products could increase the predictive ability compared with crescents alone from 0.904 to 0.944 (*p* = 0.062) with borderline significance. The model fitted best when combined with clinical or pathological parameters (including serum creatinine, urine proteinuria, and crescents) and urine complement products activation (Model 4), whose AUC was 0.973 (CI, 0.933–1) (**Figure 5**).

## Discussion

High levels of crescent formation are often associated with rapid progression of kidney disease and clinically severe proteinuria or hematuria in patients with IgAN. To date, the pathogenesis of crescent formation in IgAN is unclear. In this study, we found a strong association between urinary complement activation products and crescents on kidney biopsy, especially in those with more than 50% crescents. Additionally, the levels of urinary C4d or glomerular C4d deposition representing the lectin pathway in IgAN were strongly associated with the proportion of crescents in the kidney. These findings indicate that excess complement activation is involved crescent formation in IgAN. Urinary C4d might be a potential biomarker for monitoring crescent formation in this disease.

Based on the frequent codeposition of C3 with IgA in the glomerular mesangium, complement activation has been speculated to be an important player in the pathogenesis of IgAN since the 1980s (10, 11). Codeposition of alterative pathway components such as properdin (75–100%), factor H (30–90%), and C3 (>90%) with IgA was reported [11]. Complement activation by the lectin pathway is also involved in the progression of IgAN. MBL was found to be codeposited with IgA in the mesangial area in 17%–25% of IgAN biopsies (12, 13), and deposition of MBL or C4d by the lectin pathway correlated with the severity of the disease and could predict renal outcome (9, 14, 15). In the current study, we found that the patients with a proportion of crescents greater than 50% had much higher (10–50 times) urinary levels of activated complement products in both the alternative and lectin pathways than the other IgAN patients. In addition, these urinary complement products were associated with severe proteinuria or a low estimated glomerular filtration rate (GFR). These findings suggest that diffuse crescent formation with rapidly progressive kidney failure was associated with overactivation of both the alternative and lectin pathways. Crescent formation is partially related to lectin pathway activation. In this study, we did not find that circulating complement products were associated with crescents or clinical parameters (**Supplementary Figure 1**). These results indicate that urinary complement levels, but not circulating complement levels, represent renal complement activation. However, only a small number of patients in this study had both serum sample and urine sample, which limited the findings. Nevertheless, we found that the urinary C4d level, representing the lectin pathway, showed a linear relationship with the proportion of crescents. In addition, histological C4d-positive rate synchronously increased with the crescent percentage elevation, indicating C4d as a potential useful biomarker for monitoring crescent formation in this group of patients.

Extensive crescent formation in IgAN is often associated with rapidly progressive kidney failure and other pathologies. The KDIGO guidelines recommend aggressive immunosuppressive therapy; however, even with this approach, our prior cohort study showed that patients with more than 50% crescent formation demonstrated poor outcomes, with more than two-thirds of patients developing ESKD. To date, specific therapy for crescentic IgAN is lacking. In this study, we demonstrated that excess complement activation might involve diffuse crescent formation in IgAN, especially through the lectin pathway. This finding suggests that approaches that target complement activation (such as the recently available mannan-associated lectin-binding serine protease-2 (MASP-2) antibodies) may represent a promising option for the treatment of crescentic IgAN. To date, two case reports have shown that complement inhibition using the humanized anti-C5 monoclonal antibody eculizumab may be beneficial to crescentic IgAN (16, 17).

The definition of crescentic IgAN is still controversial. In the 2012 KDIGO guidelines, crescentic IgAN was defined as IgAN with crescents in more than 50% of glomeruli in the renal biopsy with rapidly progressive renal deterioration. In this study, we included patients with different proportions of crescent formation. We found that urinary complement activation products, including C3a, C5a, and Bb, increased significantly when the proportion of crescents reached 50% or more, while they remained at low levels and showed no difference among the different groups with a proportion of crescents less than 50%. These results indicated that crescentic IgAN with a proportion of 50% or more represents a specific phenotype.

In conclusion, in this study, we found that IgAN patients with large numbers of crescents, especially those with 50% crescent formation, demonstrated much higher levels of both alternative and lectin pathway complement activation products in urine. These findings indicate that excess complement activation is involved in crescent formation in IgAN. Thus, approaches that target complement activation may represent a promising option for the treatment of crescentic IgAN. Furthermore, urinary C4d levels were associated with the proportion of crescents on biopsy and disease severity and may be a useful biomarker for disease monitoring.

## Data Availability Statement

The raw data supporting the conclusions of this article will be made available by the authors, without undue reservation.

## Ethics Statement

The studies involving human participants were reviewed and approved by Peking University First Hospital. The patients/participants provided their written informed consent to participate in this study.

## Author Contributions

ZW and XX designed and conducted the study, analyzed the results, and prepared the manuscript. JYL, XZ, JH, and MW helped with the experiment. JCL and HZ reviewed the manuscript. All authors contributed to the article and approved the submitted version.

## Funding

This work was supported by the National Natural Science Foundation of China (81925006, 81670649, 81800639), the National Key Research and Development Program of China (2018YFC1314004), the Capital Health Development Research Project of China (2018-2-4073), Capital of Clinical Characteristics and the Applied Research Fund (Z161100000516005), and Chinese Academy of Medical Sciences Research Unit (No. 2019RU023). Grants from the Science and Technology Project of Beijing, China (D18110000011803) and CAMS Innovation Fund for Medical Sciences (2019-12M-5-046).

## Conflict of Interest

The authors declare that the research was conducted in the absence of any commercial or financial relationships that could be construed as a potential conflict of interest.

## Publisher’s Note

All claims expressed in this article are solely those of the authors and do not necessarily represent those of their affiliated organizations, or those of the publisher, the editors and the reviewers. Any product that may be evaluated in this article, or claim that may be made by its manufacturer, is not guaranteed or endorsed by the publisher.

## Supplementary Material

Supplementary Table 1 Baseline characteristics of IgAN patients with various crescents enrolled for plasma complement tests. Scr, serum creatinine.

Supplementary Figure 1 Plasma activated complement products are not always associate with the proportion of crescents in IgA nephropathy. NS., Not significant.

Supplementary Figure 2 Year distribution of urine samples recruitment.

Supplementary Figure 3 Year distribution of blood samples recruitment.

Supplementary Figure 4 Year distribution of histological samples recruitment.

Supplementary Figure 5 ROC curve of crescent in evaluation of predicting ESKD.
