# Supplementary material for: Complement Activation Is Associated With Crescents in IgA Nephropathy
Source: Front Immunol. 2021 Sep 14;12:676919. doi: 10.3389/fimmu.2021.676919 (PMC8477028; doi:10.3389/fimmu.2021.676919)
Supplement: Supplementary Table 1 — Baseline characteristics of IgAN patients with various crescents enrolled for plasma complement tests. Scr, serum creatinine. [file DataSheet_1.docx]

**Supplemental Table 1. Baseline characteristics of IgAN patients with various crescents enrolled for plasma complement tests.**

|  | 0%(N=10) | 0-24%(N=22) | 25-49%(N=18) | 50-74%(N=15) | 75-100%(N=5) | Total(N=70) | *P* value |
| --- | --- | --- | --- | --- | --- | --- | --- |
| Scr (umol/L) | 109.1(74.5-138.2) | 92.2(75.2-119.2) | 186.7(97.6-332.0) | 345.9(230.0-580.3) | 574.0(370.5-795.0) | 137.6(92.3-331.0) | <0.001 |
| Proteinuria(g/d) | 0.62(0.30-1.47) | 1.08(0.63-1.87) | 3.13(1.41-5.79) | 5.52(2.83-8.30) | 4.30(2.76-4.92) | 1.94(0.91-4.75) | <0.001 |
| Cellular+Fibrocellular crescents(%) | 0(0-0) | 8.6(5.2-14.9) | 34.8(28.6-40.7) | 58.8(53.8-66.7) | 83.3(81.5-87.5) | 28.3(6.1-53.6) | <0.001 |

Scr, serum creatinine.
