# Supplementary figures and images for: Complement Activation Is Associated With Crescents in IgA Nephropathy
Source: Front Immunol. 2021 Sep 14;12:676919. doi: 10.3389/fimmu.2021.676919 (PMC8477028; doi:10.3389/fimmu.2021.676919)

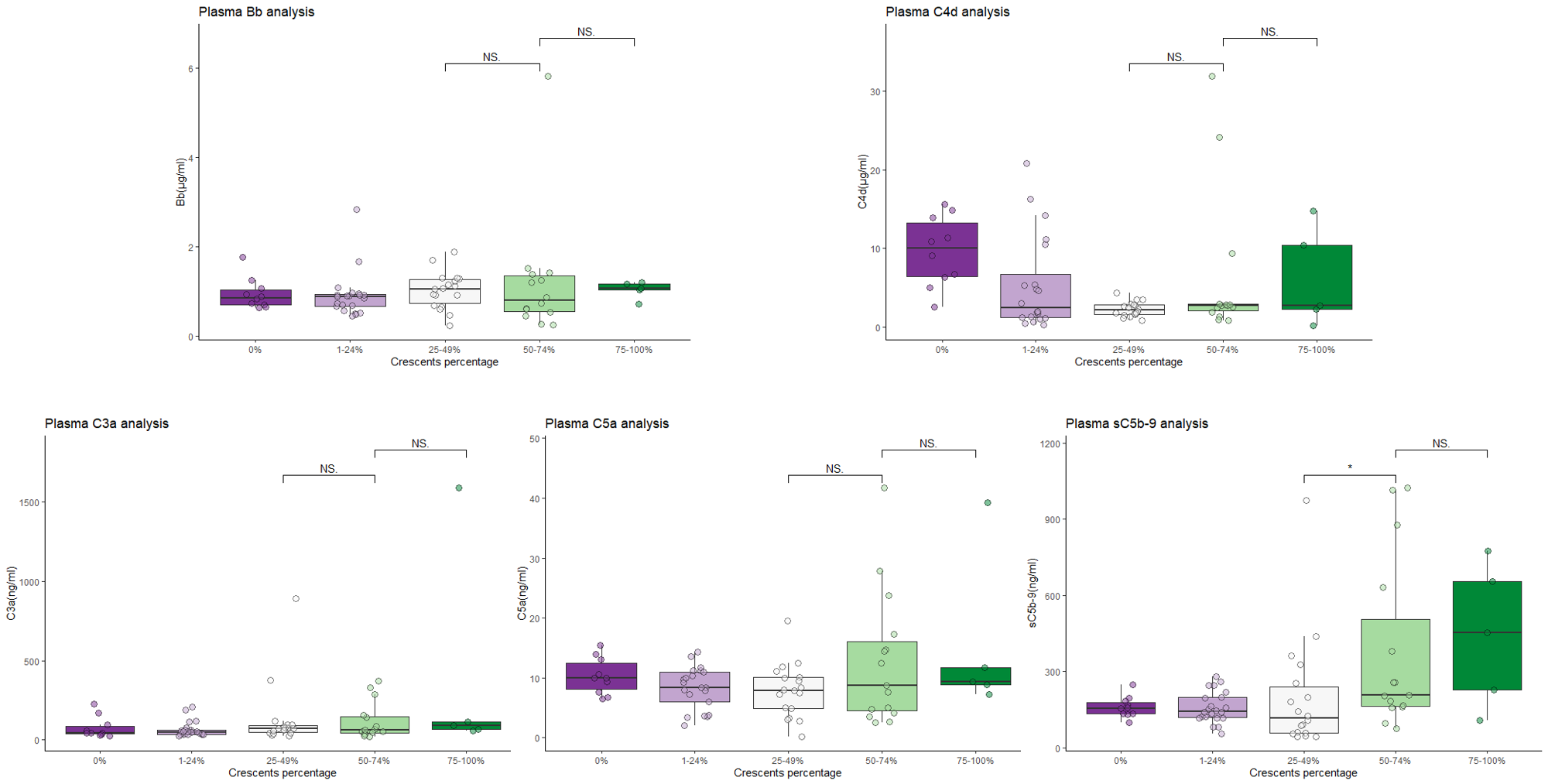

Supplement: Supplementary Figure 1 — Plasma activated complement products are not always associate with the proportion of crescents in IgA nephropathy. NS., Not significant. [file Image_1.tif]

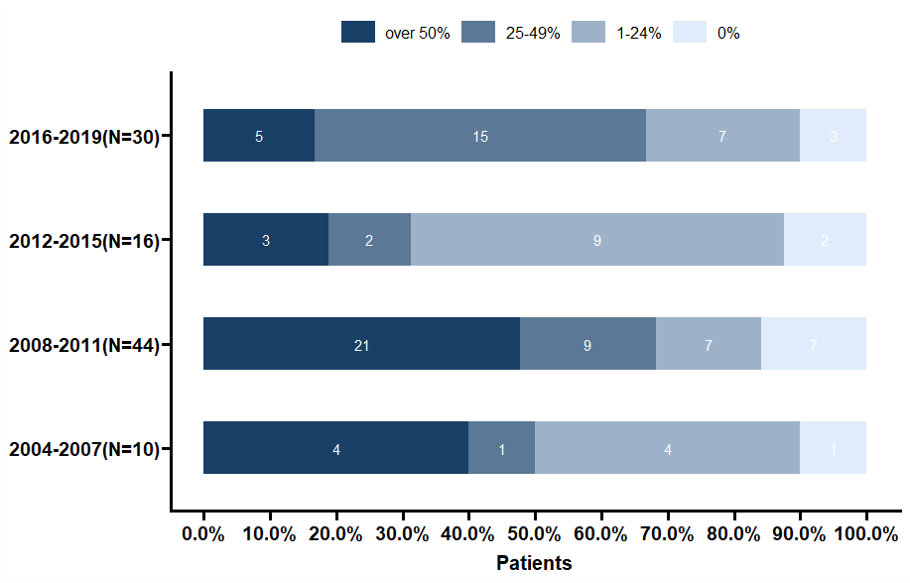

Supplement: Supplementary Figure 2 — Year distribution of urine samples recruitment. [file Image_2.tif]

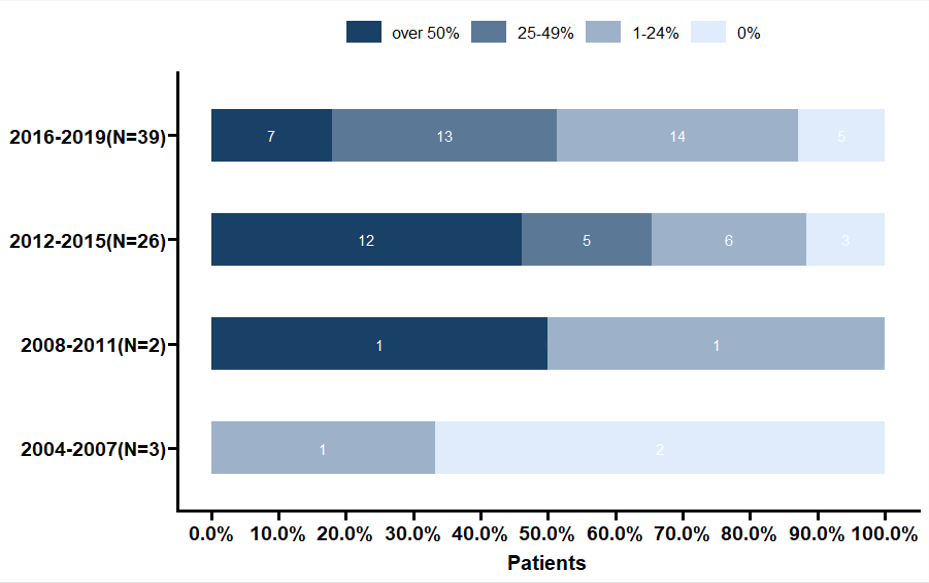

Supplement: Supplementary Figure 3 — Year distribution of blood samples recruitment. [file Image_3.tif]

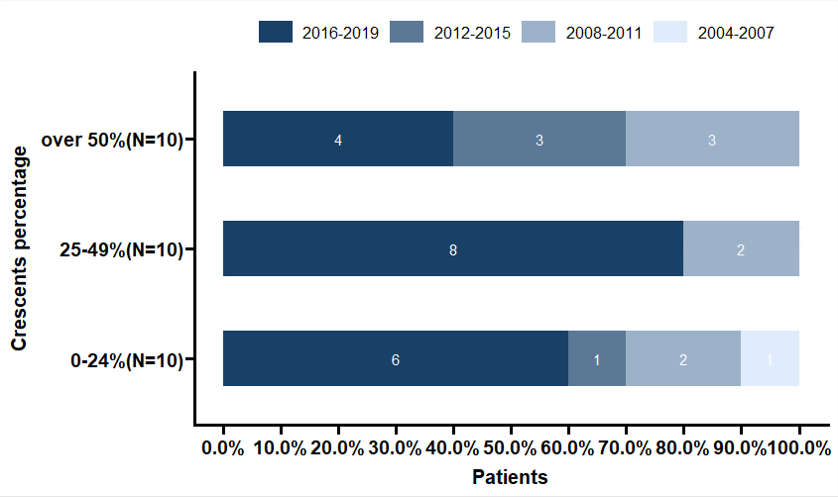

Supplement: Supplementary Figure 4 — Year distribution of histological samples recruitment. [file Image_4.tif]

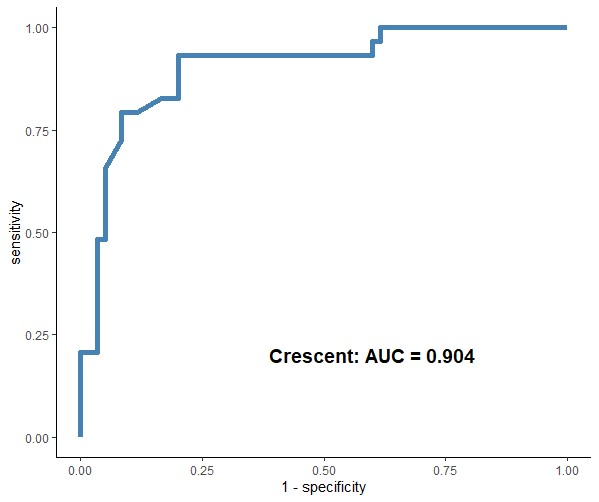

Supplement: Supplementary Figure 5 — ROC curve of crescent in evaluation of predicting ESKD. [file Image_5.jpg]
